# Intervarietal and intravarietal genetic structure in Douglas-fir: nuclear SSRs bring novel insights into past population demographic processes, phylogeography, and intervarietal hybridization

**DOI:** 10.1002/ece3.1435

**Published:** 2015-04-03

**Authors:** Marcela van Loo, Wolfgang Hintsteiner, Elisabeth Pötzelsberger, Silvio Schüler, Hubert Hasenauer

**Affiliations:** 1 Institute of Silviculture, University of Natural Resources and Life SciencesPeter Jordan Straße 82, 1190, Wien, Austria; 2 alpS-GmbHGrabenweg 68, 6020, Innsbruck, Austria; 3 Department of Forest Genetics, Federal Research and Training Centre for Forests, Natural Hazards and LandscapesHauptstr. 7, 1140, Vienna, Austria

**Keywords:** Douglas-fir, genetic diversity, genetic structure, intervarietal hybridization, refugia, varieties

## Abstract

Douglas-fir (*Pseudotsuga menziesii*) is one of numerous wide-range forest tree species represented by subspecies/varieties, which hybridize in contact zones. This study examined the genetic structure of this North American conifer and its two hybridizing varieties, coastal and Rocky Mountain, at intervarietal and intravarietal level. The genetic structure was subsequently associated with the Pleistocene refugial history, postglacial migration and intervarietal hybridization/introgression. Thirty-eight populations from the USA and Canada were genotyped for 13 nuclear SSRs and analyzed with simulations and traditional population genetic structuring methods. Eight genetic clusters were identified. The coastal clusters embodied five refugial populations originating from five distinct refugia. Four coastal refugial populations, three from California and one from western Canada, diverged during the Pleistocene (56.9–40.1 ka). The three Rocky Mountain clusters reflected distinct refugial populations of three glacial refugia. For Canada, ice covered during the Last Glacial Maximum, we present the following three findings. (1) One refugial population of each variety was revealed in the north of the distribution range. Additional research including paleodata is required to support and determine whether both northern populations originated from cryptic refugia situated south or north of the ice-covered area. (2) An interplay between *intravarietal* gene flow of different refugial populations and *intervarietal* gene flow by hybridization and introgression was identified. (3) The Canadian hybrid zone displayed predominantly introgressants of the Rocky Mountain into the coastal variety. This study provides new insights into the complex Quaternary dynamics of this conifer essential for understanding its evolution (outside and inside the native range), adaptation to future climates and for forest management.

## Introduction

Within the native distribution range of a tree species, the contemporary genetic structure provides insights into a species’ demographic history, colonization events as well as past or present hybridization events (Godbout et al. 2008; Cullingham et al. 2012; Semerikov et al. 2013). As a result of population history and complex interactions with the environment numerous forest tree species differentiated into subspecies, varieties and ecotypes (e.g., *Abies lasiocarpa, A. grandis, A. concolor, Larix sibirica, Pinus radiata*) with hybrids and introgressed individuals in zones of contact (Hunt 1993).

Douglas-fir (*Pseudotsuga menziesii* (Mirb.) Franco) is a tree species of temperate and boreal forests with both a wide native distribution range and a large artificial range outside its natural distribution, where it regenerates naturally (Fussi et al. 2013). It is a common northwestern American conifer covering a large latitudinal (19° to 55°) and elevation (sea level to 3260 m) range (Hermann and Lavender 1990; Kleinschmidt and Bastien 1992). Within its native range, two varieties are commonly recognized: the coastal variety (*P. menziesii* var. *menziesii*) and the interior variety (*P. menziesii* var*. glauca*) (Eckenwalder 2009). The native distribution area of var. *menziesii* covers the 2200 km coastal range from central British Columbia (BC) into central California. The var. *glauca* extends 4500 km from central BC across the Rocky Mountains into southern Mexico. Both varieties differ morphologically and ecologically. The var. *menziesii* grows faster and is considerably larger in size than the var. *glauca,* which is more shade tolerant, more cold-hardy, and more drought resistant (Eckenwalder 2009). The phylogeography within this species was refined by sequencing chloroplast DNA (cpDNA), which is paternally inherited in the Douglas-fir. The sequencing revealed three distinct phylogenetic lineages: the coastal, Rocky Mountain and Mexican lineage. Consequently, the var*. glauca* and its range were divided into the Rocky Mountain lineage (northern Rockies to southern New Mexico) and the Mexican lineage (central Mexico to Oaxaca) (Li and Adams 1989; Wei et al. 2011; Adams and Stoehr 2013).

**Figure 1:**
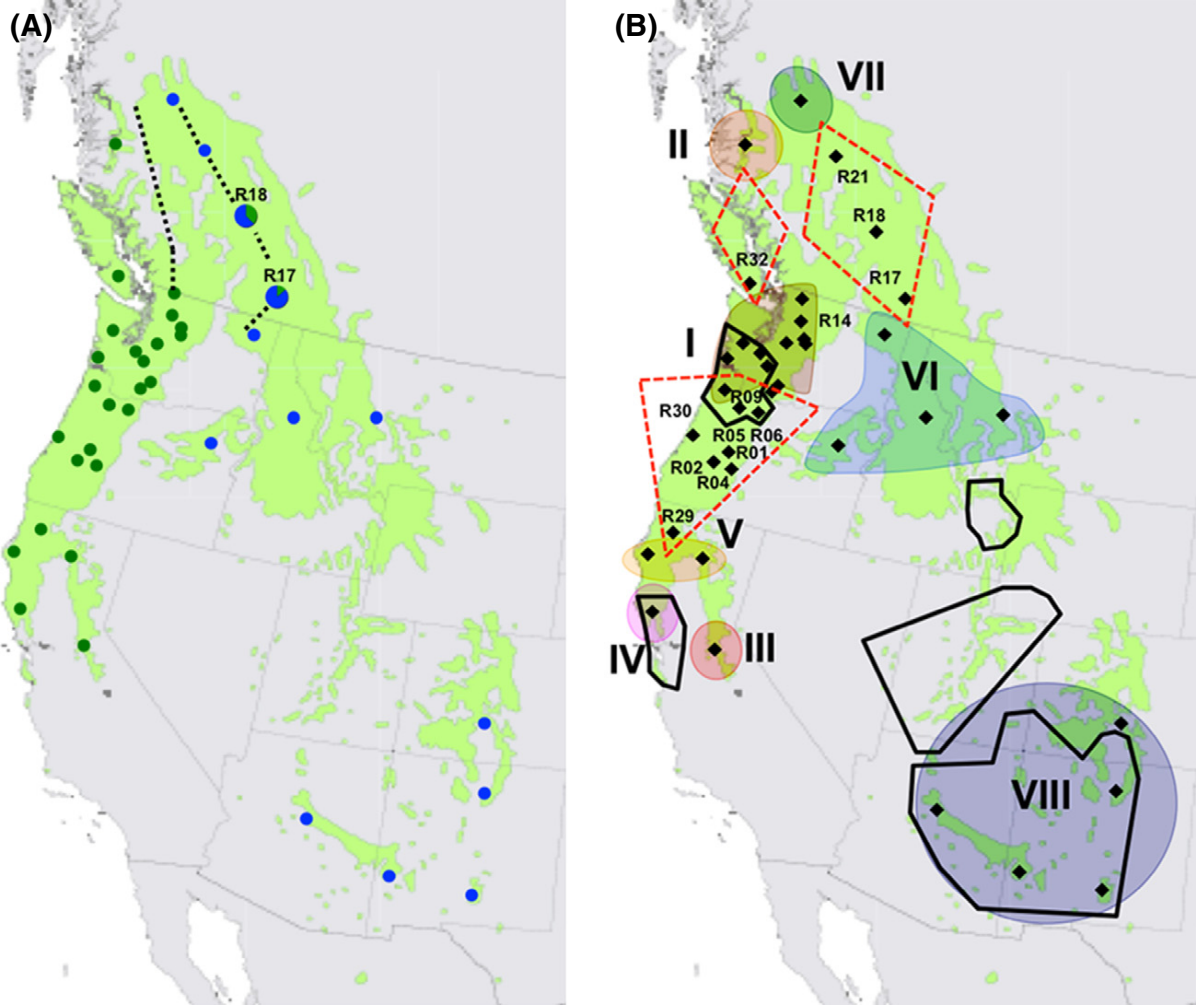
Distribution maps of Douglas-fir populations with their assignment to a variety (A), or to an intravarietal genetic cluster (B) as estimated by STRUCTURE. Populations R17, and R18 (map A) represent populations where individuals of both varieties, coastal (green color) and Rocky Mountain variety (blue color), were present. Dotted lines indicate the borders of the assumed intervarietal hybrid zone (von Rudloff 1973; Li and Adams 1989; Gugger et al. 2010; Wei et al. 2011). Map (B) displays eight intravarietal genetic clusters (I–VIII). Cluster-admixed populations (0.2 < *Qvar *< 0.8) are situated in overlapping (red dashed line) between two clusters I-II, I-(III+IV+V), and VI-VII. Black solid lines indicate glacial refugia based on fossil data detected by Gugger et al. (2010).

Divergence estimates of Douglas-fir in its three main lineages (with the Rocky Mountain lineage being the oldest) differ among recent studies (Gugger et al. 2010, 2011; Wei et al. 2011). Wei et al. (2011) dated this split to have occurred from late Miocene and through the Pliocene (10–3.2 Ma), whereas Gugger et al. (2010, 2011) reported estimates from 4.372 Ma to 755 ka (from Pliocene to mid-Pleistocene). In more detail, the split of the coastal lineage from the Rocky Mountain lineage was dated back to 8.5 Ma (Wei et al. 2011) and to 2.11 Ma (Gugger et al. 2010), respectively. Through Miocene to Pliocene, western northern America experienced a series of complex geological events such as the orogeny of mountain ranges (the Sierra Nevada and Cascades), which was probably the key factor in the vicariance initiating the varietal difference between the coastal and Rocky Mountain lineage (from here on the term variety will be used for both lineages) (Gugger et al. 2010, 2011; Wei et al. 2011). Before the Last Glacial Maximum (LGM, or Late Wisconsin, 18ka), both varieties occupied distinct glacial refugia (Gugger and Sugita 2010; Gugger et al. 2010; Wei et al. 2011). Both fossil and marker based data show dissimilarities concerning the number of the last glacial refugia for both varieties. For the coastal variety, two refugia located within the current distribution area in the USA (on the Pacific coast in Oregon/Washington (OR/WA) and in California were strongly suggested by fossil records (Gugger and Sugita 2010) (Fig.1B). Their existence was also mirrored in the phylogeographic pattern found in the terpenoids (Snajberk and Zavarin 1976). However, the two-refugium theory is in discordance with range-wide allozyme (Li and Adams 1989) or recent mitochondrial DNA (mtDNA) and cpDNA phylogeographies (Gugger et al. 2010; Wei et al. 2011), which support the one refugium hypothesis. In contrast to the coastal variety with one or two possible refugia, several refugia were proposed for the Rocky Mountain variety. The existence of three refugia situated in the Rocky Mountains of the USA was well supported by both fossil and molecular data and coalescent tests (Gugger and Sugita 2010; Gugger et al. 2010). The occurrence of other (at least 4 additional) refugia was proposed based on distribution of private cpDNA types (Gugger et al. 2010). In addition to all these refugia situated south of the Laurentide ice sheet during the LGM and within the current distribution range, Wei et al. (2011) proposed one possible glacial refugium of this variety being set outside the current distribution range, in unglaciated areas to the north of the Laurentide ice sheet.

The postglacial colonization from refugia after the LGM led to contact zones and hybridization events between the coastal and Rocky Mountain varieties along the eastern Cascades of Oregon and Washington and in the interior of BC in Canada, where a broad hybrid zone was formed (Eckert et al. 2009; Gugger et al. 2010; Wei et al. 2011).

Given the genetic complexity of the Douglas-fir (characterized by the presence of different varieties, migration from several different refugia after the LGM, intervarietal hybridization, expected interrefugial gene flow and possible existence of a northern glacial refugium outside the present distribution area), this study aimed at investigating the geographical genetic structure at the intervarietal level as well as at the level within coastal and Rocky Mountain varieties separately in order to obtain a more detailed picture on the phylogeographic pattern, selected historical demography, intervarietal hybridization and introgression patterns of Douglas-fir. Instead of using new and/or a larger number of dominant organelle loci (cpDNA and mtDNA) than used in previous range-wide phylogeographic and demographic studies (e.g., by Gugger et al. (2010) and Wei et al. (2011), we followed the recommendations of these previous studies as well as those of Eckert et al. (2009), to use biparentally inherited (codominant) nuclear DNA markers for this species. Consequently, we employed 13 highly variable nuclear microsatellite markers (nuSSRs) for genotyping 38 populations situated in the USA and Canada. The other reasons we selected these markers is that they allow Douglas-fir variety identification (Fussi et al. 2013) and the study of hybridization/introgression events in general (Lexer et al. 2010; Cullingham et al. 2012; Smith et al. 2013). Owing to recombination, many realizations of the demographic process can be retrieved with nuclear markers, reducing the variability of the estimates of population parameters (Lascoux et al. 2004).

We compared and evaluated the phylogeographic genetic structure revealed by SSRs with the previously identified and proposed glacial refugial history and addressed three questions on the Quaternary glacial history and colonization of Douglas-fir: (1) Did the coastal variety expand from (at least two) different glacial refugia located in the current distribution range; (2) which refugia contributed to the spread of the investigated Rocky Mountain variety after the LGM; and (3) did intervarietal F1 hybrids rather than backcrosses to one of the variety exist within analyzed populations of the hybrid zone in BC?

## Materials and Methods

### Sampling

A total of 766 individuals representing 38 populations from the USA and Canada and covering the natural distribution range of the coastal and Rocky Mountain variety were collected (Table S1A and Fig. S1). Higher numbers of populations were chosen to cover specific areas in OR and WA which are recommended for seed transfer to the majority of European countries (Breidenstein et al. 1990). A minimum of 20 individuals per population were used except for one population where samples from 18 individuals were collected. For 21 populations, cambium samples were obtained from Austrian provenance trials which were established by the Federal Research and Training Centre for Forests, Natural Hazards and Landscapes (Vienna, Austria) in the late 1970s using seeds from the controlled IUFRO-seed collection section (Barner 1973), or from seeds collected in natural populations by employees of the institution. For the other 17 populations, seeds were taken from collections of the USDA Forest Service-Placerville Nursery, the BC Forest Service and from R. Klumpp (University of Natural Resources and Life Sciences, Institute of Silviculture, Vienna, Austria). These were cultivated in a nursery for 2–3 months until the seedlings were approximately 5 cm in height. Both cambium samples and whole seedlings were dried in silica gel prior to the DNA extraction. The number of mother trees, the used seed collections and provenance trials originate from, are listed in the Table S1A.

### DNA extraction and genotyping

The DNA was extracted from needle or cambium tissue using an OMEGA E.Z.N.A Plant DNA Kit (OMEGA Bio-Tek, Inc., Norcross, Georgia, USA) according to the manufacturer’s instructions. The quality and concentration of the extracted DNA were measured on a 1.5% agarose gel electrophoresis and the Implen OD600 DiluPhotometer. For PCR reactions, the DNA was subsequently diluted with PCR water to an equal concentration of 30 *η*g/*μ*L. A total of 17 highly polymorphic dinucleotide nuSSRs were selected from Slavov et al. (2004). These were tested on their performance (yield and specificity) on a set of 16 individuals. The finally selected 13 nuSSRs (PmOSU_2G12, PmOSU_4A7, PmOSU_3B2, PmOSU_5A8, PmOSU_2D4, PmOSU_1F9, PmOSU_3F1, PmOSU_2D6, PmOSU_1C3, PmOSU_2C2, PmOSU_3B9, PmOSU_3D5, PmOSU_2D9), which allowed clear-cut scoring and amplified one locus only, were further tested for their use in multiplex PCRs with a final arrangement into four multiplex PCR combination groups (Table S2). For simplicity, the prefix “PmOSU” is going to be omitted from now on. The selected nuSSRs reside on eight different linkage groups with the shortest distance of 15.6 (cM) between 2D9 and 3B2 from the linkage group 1 (for further details see Slavov et al. 2004). PCR amplifications were performed with the QIAGEN Type-it Microsatellite PCR Kit following the manufacturer’s protocol and annealing temperature resulting from the testing (Table S2). The PCR amplifications of the multiplex PCR combination 4 turned out to be ineffective in the majority of individuals originating from five Rocky Mountain populations and consequently resulted in missing values. These PCRs were therefore repeated using a touchdown procedure with annealing temperatures of 55–45°C for 10 cycles, each 90 sec, followed by 30 cycles at 50°C, each 60 sec. NuSSRs genotypes were resolved on an ABI PRISM™ 3100 DNA Genetic Analyzer (Applied Biosystems. Inc., Foster City, California, USA). The sizing of fragments was carried out with a Genescan 3.7 and Genotyper® 2.0 software (Applied Biosystems), utilizing the internal GENESCAN™-500 ROX™ Size Standard (Applied Biosystems). Prior to performing any statistics, the genotypes were checked and corrected for null alleles and scoring errors by MICRO-CHECKER 2.2.3 using the “Brookfield 1” algorithm (Van Oosterhout et al. 2004).

### Genetic diversity and differentiation

Standard descriptive genetic diversity statistics was estimated at both the locus and the population levels. Microsatellite loci of both varieties were characterized for the number of alleles (*N*_a_), mean number of alleles (*A*), effective number of alleles (*N*_e_), observed heterozygosity (*H*_O_), expected heterozygosity (*H*_E_), inbreeding coefficient (*F*_IS_), and allelic richness (*A*_S_). They were corrected for sample size by rarefraction, with the software FSTAT v. 2.9.3.3 (Goudet 1995, 2001) and the GenAlEx v. 6.5 software by Peakall and Smouse (2006, 2012). At the population level, the descriptive statistics included calculations of allelic richness (*A*_S_) using the approach of Szpiech et al. (2008), and estimations of expected heterozygosity (*H*_E_), observed heterozygosity (*H*_O_), and inbreeding coefficient (*F*_IS_) using the GenAlEx v. 6.5 software (Peakall and Smouse 2006, 2012). Finally, the Hardy–Weinberg (HW) exact tests were carried out for both levels (population and locus) to estimate the heterozygote deficiency with the software GENEPOP v. 4.1.4, using the default values (Raymond and Rousset 1995; Rousset 2008).

### Genetic structure and clustering

The genetic structure was analyzed at two levels, at the intervarietal level, and at the intravarietal level. For the former, analyses of genetic structure were run on the entire dataset including both varieties, whereas for the latter all analyses were run on data subsets representing populations either of the coastal variety or the Rocky Mountain variety. The populations of the whole dataset were divided into two variety subsets (*K* = 2) using Bayesian admixture analysis in STRUCTURE v.2.3.4. (Pritchard et al. 2000; Falush et al. 2003, 2007). Twenty replicates of a run with a burn-in period of 50,000 and Markov chain Monte Carlo (MCMC) iterations of 100,000 were carried out based on the admixture model with correlated allele frequencies (Falush et al. 2003, 2007). These selected adjustments for the burn-in time and MCMC iterations resulted from tests optimizing the trade-off between precision and simulation time. STRUCTURE HARVESTER (Earl and von Holdt 2012) was used to generate an input file for the software CLUMPP v.1.1.2 (Jakobsson and Rosenberg 2007), which was used to estimate the mean variety-membership coefficient (*Qvar*) of each individual and population. An individual/population was declared as coastal with *Qvar* (membership coefficient) > 0.9, Rocky Mountain with *Qvar* < 0.1 and intervarietal admixed with 0.9 > *Qvar* > 0.1.

The genetic population structure of both varieties was inferred using two different clustering approaches (Bayesian analysis of STRUCTURE and principal coordinate analysis [PCoa]). The Bayesian STRUCTURE analysis was run on both variety subsets separately using identical settings as described before. In addition, each subset was cleaned from possible intervarietal-admixed individuals (0.9 > *Qvar* > 0.1) as well as from individuals of the opposite variety when present. Each *K* was run 20 times. As Δ*K* (the real number of clusters) detects the uppermost level of genetic structure where several hierarchical levels exist (Evanno et al. 2005), it was rational to carry out a hierarchical STRUCTURE analysis within both variety subsets as these represent populations originating from different refugia and might also represent populations mixed between two refugia in the zone of contact. Thus, the size of each variety subsets was reduced step by step according to the significance of the results as inferred with hierarchical analyses of the Δ*K* method (Evanno et al. 2005). Means of estimated intravarietal membership coefficient (*Qintra*) for each individual and population of the 20 runs per *K* were computed with the software CLUMPP v.1.1.2 using the Greedy option and random input orders (Jakobsson and Rosenberg 2007). The means were then plotted using DISTRUCT v.1.1 (Rosenberg 2004). The program STRUCTURE HARVESTER (Earl and von Holdt 2012) was used to estimate Δ*K* within the analyzed dataset. Subsequently, each population was assigned to the cluster for which its inferred individual and population ancestry (*Qintra*) was the highest. This assignment was also checked visually on DISTRUCT plots in order to evaluate the estimated *Qintra* value of populations and their composition. Although running STRUCTURE on a population level underestimates the actual number of clusters within the dataset, we preferred this strict and controllable procedure over reshuffling the individuals among clusters, which might overestimate the number of existing clusters and cause assignment problems especially for populations resulting from gene flow between refugia. If *Qintra* for one or more populations within the studied dataset was 0.2 < *Qintra* < 0.8, the procedure was additionally rerun in STRUCTURE under the *locprior* using the Δ*K* value recommended by Evanno (Evanno et al. 2005). The remaining settings were identical to those described previously. The use of the *locprior* option is of significance when detecting genetic structures at lower levels of divergence, as expected for the coastal variety (Li and Adams 1989; Krutovsky et al. 2009) with no false positives if there is no structure present (Hubisz et al. 2009). We did not attempt to force individual populations to belong to a particular cluster as we expected that some of them might be of admixed nature because of refugia-mixed and/or variety-mixed origin. Therefore, whenever the *locprior* option led to identical results (in the assignment of populations to a cluster and/or their admixed nature) to those without this option, the assignment of populations was accepted. Populations with 0.2 < *Qintra* < 0.8 were assigned to reflect cluster-mixed populations.

Subsequently, pairwise Jost’s *D* values among all estimated STRUCTURE clusters were calculated and tested for significance using permutations in GeneAlEx 6.5 (Peakall and Smouse 2006, 2012). Jost’s *D* (Jost 2008) is more precise in quantifying differentiation when applying highly polymorphic markers such as SSRs (Meirmans and Hedrick 2011).

As a second approach, population clustering and the relations between populations were calculated with a pairwise codominant genotypic distance followed by a covariance standardized PCoa using GeneAlEx 6.5 (Peakall and Smouse 2006, 2012). This clustering was performed for the whole dataset as well as for the two variety subsets separately using both cleaned and not cleaned datasets from intervarietal-admixed individuals and from individuals of the opposite variety. The analyses with and without the cleaned data allow estimating the influence of intervarietal-admixed individuals on the clustering. In addition to this, we tested the clustering of populations represented by intervarietal-admixed individuals only. To do so, we run PCoa with the cleaned data of the entire whole set supplemented by “new” populations. These “new” populations were built using the intervarietal-admixed individuals (with 0.4 < *Qvar* < 0.6). In more detail, intervarietal-admixed individuals of R18, R21, and R39 were used to assemble such new populations a18, a21, and a39.

### Intervarietal and intravarietal demographic history

Before we estimated the demographic history of genetic clusters revealed by preceding PCoa and STRUCTURE analyses, the divergence time of both coastal variety and Rocky Mountain variety was estimated. We did it by applying Approximate Bayesian Computation (ABC) analysis in DIYABC, version 2.0 (Cornuet et al. 2014) to all nuSSR loci. The intervarietal divergence was estimated using populations of the coastal (R01-R11, R16, R30, Fig. S1) and Rocky Mountain (R22-R26, Fig. S1) variety. These populations overlap with areas where Miocene and Pleistocene fossils of Douglas-fir and a refugium for each variety characterized by cpDNA were detected (Gugger et al. 2010). Thereafter, in order to clarify possible refugial origin of the discovered genetic clusters, different putative historical scenarios were tested and dated following the standard protocol of DIYABC. We were aware of the fact that a distinct genetic cluster does not immediately represent a refugial population. The analyzed populations of each cluster are shown in Table S7. In all runs, 1,000,000 datasets were simulated for each scenario. A total of 1% of the simulated datasets most similar to the observed data were used to estimate the relative posterior probability (with 95% confidence intervals [CIs]) of each scenario via logistic regression and posterior parameter distributions according to the most likely scenario. Because DIYABC version 2.0 selects the optimized rate from a given mutation range, we assigned mutation rates of nuSSRs to vary between 10^−4^ and 10^−2^ and thus to correspond to a general mutation rate for nuSSRs (Miah et al. 2013). All intervarietal-admixed individuals were omitted from these analyses. Following parameters were estimated: divergence time (*t*), effective population size of each population (*N*_1_, *N*_2_), and effective population size of ancestor (*N*_a_). As *t* was expressed in the number of generations, we used an approximate average generation time of 100 years to convert it into years, following Gugger et al. (2010, 2011).

### Hybridization and introgression pattern in intervarietal-admixed populations in BC (Canada)

In order to assess whether intervarietal F1 genotypes or backcrosses exist within analyzed populations of the hybrid zone in BC (Canada), all intervarietal-admixed individuals with 0.2 < *Qvar* < 0.8 as estimated in STRUCTURE analysis (*K* = 2) from studied populations in BC have been selected. Subsequently, values of the intervarietal heterozygosity (*IH*) and maximum likelihood hybrid index (*HI*) were estimated following the approach as described by Lexer et al. (2010). In more detail, we tested whether genetically admixed intervarietal individuals, previously identified by the STRUCTURE analysis, were more compatible with the simulated F1 individuals (maximum *IH* and intermediate *HI*), or represent rather recombinant hybrids and introgressants of subsequent generations (reduced *IH* and *HI* values moving toward values of the coastal or Rocky Mountain variety). In the analysis, genotypes of 500 F1 individuals were simulated with the HYBRIDLAB software v.1.1 (Nielsen et al. 2006) using 32 randomly selected individuals from a coastal variety subset with *Qvar *> 0.99, and 32 individuals of the Rocky Mountain variety subset with *Qvar* < 0.01. Then, calculations of *IH* and *HI*, including 95% CIs, were carried out and plotted for simulated and observed intervarietal-admixed individuals and parentals with R package Introgress (Gompert and Buerkle 2010) following Gompert and Buerkle (2009).

## Results

### Genetic diversity and differentiation

The majority of nuSSRs were variable with up to 75 alleles (*N*_a_) observed per locus and variety (Table S3). The locus 5A8 had the lowest number of alleles represented by 13 different alleles for the coastal and nine for the Rocky Mountain variety. The observed (*H*_O_) and expected (*H*_E_) heterozygosities varied from 0.255 to 0.924 among 13 nuSSR loci (Table S3), and from 0.491 (R24) to 0.904 (R11) among populations for all loci (Table S1B). Both heterozygosities were highest in the population R11 (WA), which is a population of the coastal variety (Table S1B). The lowest values for *H*_O_ and *H*_E_ were found in the population of the Rocky Mountain variety R24 (New Mexico) located in the southernmost area of this variety analyzed in the present study. This population exhibited 100% missing values for three loci (2D6, 3F1, 5A). The highest values of *As* (6.5) were found in four populations of the coastal variety. The smallest values for allelic richness (*As* = 5.4 or 5.7) were calculated in populations of both varieties with the northernmost (R38, R39) or the southernmost (R34, R22-R24) distribution in the studied native range. The inbreeding coefficients (*F*_IS_) were significantly positive indicating deviations from Hardy–Weinberg expectations at both population level, and also on the locus level within variety (Table S1B, Table S3). This result (1) is most likely caused by a high frequency of null alleles, allelic dropout in the nuSSR loci, and the large polymorphism and (2) is consistent with all previously published data on Douglas-fir analyzed using SSRs. In this data, individuals of a population were represented by trees or seeds collected in close or large distances (Krutovsky et al. 2009; Fussi et al. 2013). We minimized the problem with null alleles using the MICRO-CHECKER software.

### Genetic structure and clustering

The genetic structure analysis performed by the software STRUCTURE allowed us to assign populations to both varieties (Fig.1A). Except for four populations (R17, R18, R21, R39), all populations were clearly assigned either to the coastal variety with *Qvar* of > 0.9 or the Rocky Mountains variety with *Qvar* < 0.1 (Table S1B). Within the four populations, genotypes of both varieties (R17, R18) and/or larger numbers of intervarietal-admixed genotypes (R17, R18, R21, and R39) were present. Consequently, the whole dataset was divided into two variety subsets representing the coastal and the Rocky Mountain variety with 25 (R01-R16, R19, R29, R30, R32, R34-R38) and 13 populations (R17, R20-R28, R33, R39), respectively (Fig.1A).

In the hierarchical STRUCTURE analysis, the coastal variety subset was divided into three clusters, a northern (R38), a central (R03, R07-08, R10-13, R15-16, R19) and a southern cluster (R34-37). Nine populations (R01, R02, R04-R06, R09, R14, R29, and R30) represented cluster-mixed populations between the central and southern cluster and two (R14, R32) between central and northern cluster (Fig.1B). The southern cluster was further subdivided into three clusters (III–V) with the populations R34, R35, and R36-37 (Fig.1B, Table S2B). In total, five geographically and genetically distinct coastal clusters (I–V) were detected including three in California, one in BC and one between these two regions (Fig.1B, Table S1B).

When compared to the coastal variety, the Rocky Mountain variety split into a smaller number of genetic clusters. Its 13 populations were divided into two clusters, cluster VIII (R22-R26) situated in the south of the Rocky Mountains and a cluster with the remaining populations (Fig.1B, Table S2B). The latter was subsequently divided into clusters VI and VII. The populations R20, R27, R28, and R33 of cluster VI are distributed in the area of the Rocky Mountains in the north of the USA and the southeast of BC. The cluster VII lies to the far north of the distribution area of the species in BC and contains only the population R39. The populations R17, R18, and R21 were cluster-mixed populations between cluster VI and VII. Results of cluster heterozygosities (*H*_O_ and *H*_E_) were similar to those calculated for populations. The highest values of *H*_O_, *H*_E_, and *A*s were found in the populations of cluster I, located in the northern area of Oregon and the south of Washington to the west of the Cascades. From cluster I, a decline of diversity (*H*_E_ and *A*s) was found toward the southernmost cluster III and the northernmost cluster II within the coastal variety. The same is true for the Rocky Mountain variety when comparing the values *H*_E_ and *A*s of the cluster VI with the cluster VII in the north and the cluster VIII in the south (Table S1B). As mentioned previously, we aimed for discovering robust clusters and not for the largest number of them. Nevertheless, trends of further substructuring were evident within clusters I, VI, and VIII (data not presented).

Pairwise calculated Jost’s *D* differentiation values among all clusters estimated by the hierarchical STRUCTURE analysis were significantly different displaying larger values in intervarietal than in intravarietal comparisons (Table S4). For the calculations, 12 SSRs were used except the locus 5A8 which largely exhibited no amplifications (thus missing data) in populations of Rocky Mountain cluster VIII. In the intravarietal comparisons, genetic clusters isolated by largest geographic distances were at the same time the most genetically differentiated. In more detail, the largest pairwise Jost’s estimates were found between the coastal variety clusters II and III with 0.580 and between the Rocky Mountain clusters VII and VIII with 0.667, respectively.

**Figure 2:**
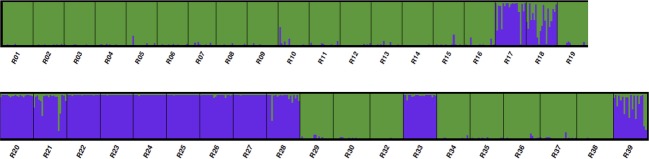
STRUCTURE results plotted by DISTRUCT for genetic structure at the inter varietal level (*K* = 2) for 766 individuals representing 28 populations (marked by R) of the coastal (green color) and Rocky Mountain variety (blue color). Individuals are grouped by populations.

**Figure 3:**
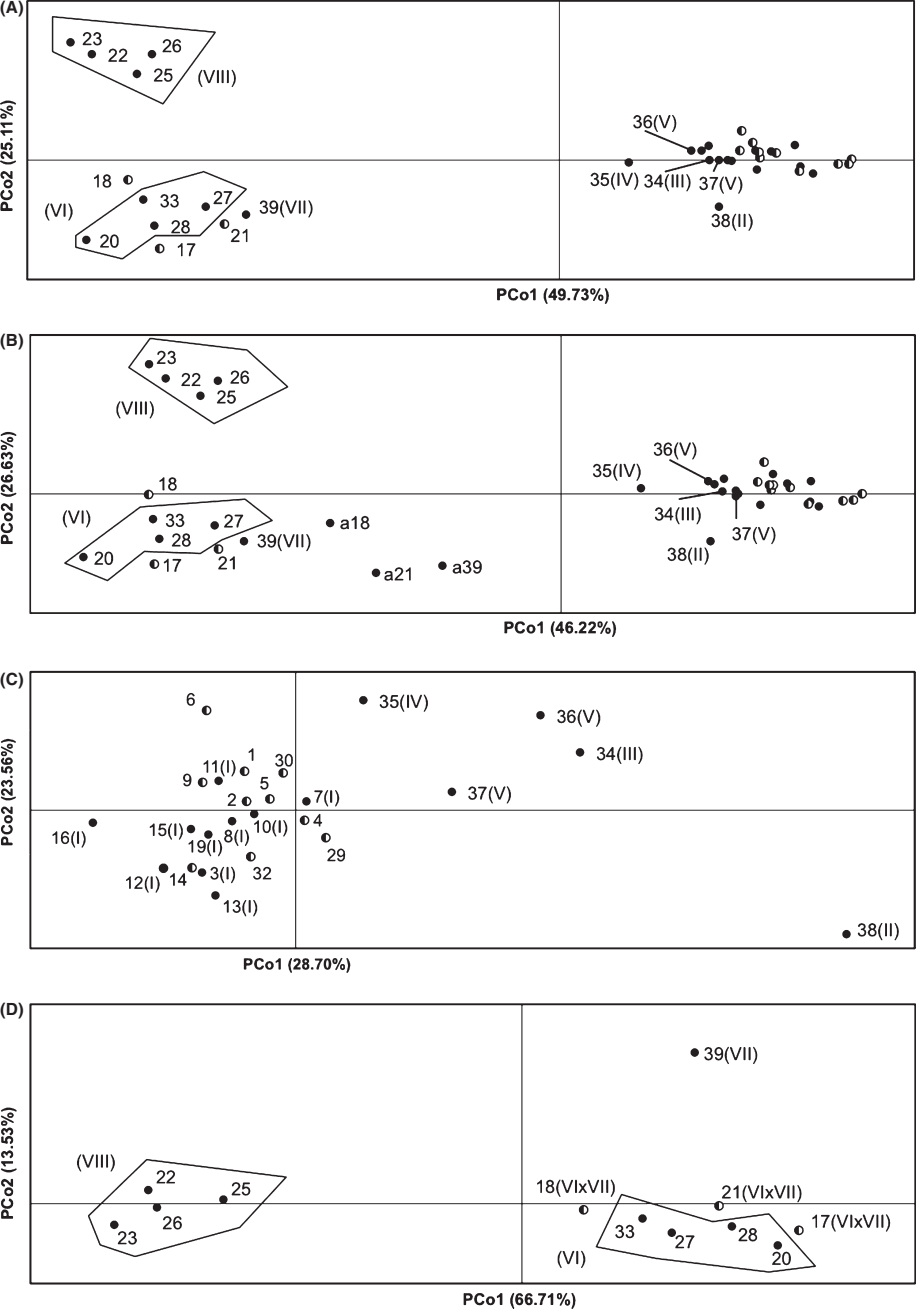
Plots of principal coordinates analysis (PCoa) described by two principal coordinates (PCo1, PCo2) and explained genetic variance (%) for populations (R01-R39) without intervarietal-admixed individuals and individuals of the opposite variety for the entire dataset (A) for the entire data set and three “new” populations (a18, a21, a39) consisting of intervarietal-admixed individuals only (B), for the coastal variety (C) and the Rocky Mountain variety (D). Roman numbers (in parentheses) at the back of each population represent a membership to the particular genetic cluster as assigned by STRUCTURE analysis. Populations of identical STRUCTURE cluster are grouped together (marked areas). The cluster-admixed populations are embodied by half-filled dots. Dots with missing population numbers represent populations of the cluster I and its cluster-admixed populations.

For all PCoa, we excluded the population R24 from datasets as its missing allele values in three loci could influence the calculated genetic distances. In the plots of PCoa, the presence of the individuals of the opposite variety as identified in the populations of the Rocky Mountain variety in Canada (Fig.2) as well as the presence of intervarietal-admixed individuals within the different datasets did not influence the clustering (Fig.3A–D, Fig. S2A–C). More specifically, the number of clusters, their distribution within the PCoa plots, and even the position of all but one (R18) populations within the plots (Fig.3A and Fig. S2A) were similar for both cleaned and not cleaned datasets. As expected, populations represented only by intervarietal-admixed individuals (a18, a21, a39) were located in between the Rocky Mountain and the coastal variety, but closer to the Rocky Mountain variety (cluster VI and VII) of which populations they were derived from (Fig.3B).

The PCoa at the intervarietal level in both datasets (cleaned and not cleaned) revealed more structure than the Bayesian analysis by STRUCTURE at this level. In total, three clusters were separated; two of the Rocky Mountain variety and one of the coastal variety with two populations (R35 and R38) clearly detached from the rest of this cluster (Fig.3A). The first two PCoa axes accounted for 74.84% of genetic variance.

At the intravarietal level of the coastal variety, the Californian clusters (III, IV, and V) and the northern cluster II (R38) in BC were separated from the rest (Fig.3C). The first two axes accounted for 52.25% of genetic variance. The PCoa of Rocky Mountain Douglas-fir clearly divided the northern cluster VII from the residual clusters VI and VIII (Fig.3D). The first two principal components accounted for a larger fraction of the genetic variance (80.24%) within the Rocky Mountain variety when compared to populations of the coastal variety. All – but the R14 – cluster-admixed populations of the coastal and the Rocky Mountain varieties were positioned between matching clusters (Fig. S2B and C, Fig.3C and D) and were thus in line with the results of STRUCTURE.

### Intervarietal and intravarietal demographic history

**Figure 4:**
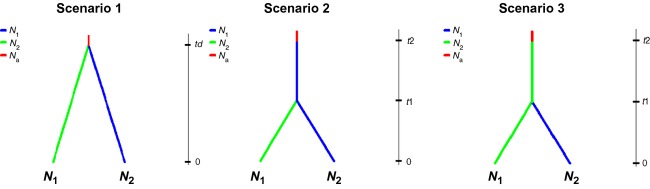
Three historical scenarios tested for genetic clusters of the Douglas-fir in an Approximate Bayesian Computation (ABC) as implemented in DIY ABC (Cornuet et al. 2014).

Intervarietal divergence between coastal and Rocky Mountain varieties with a generation time of 100 years was estimated to have occurred 708 ka ago (95% CI: 984–316 ka). To estimate and to clarify the intravarietal demographic history of the eight genetic clusters revealed by STRUCTURE and PCoa, both varieties were treated separately. We designed putative historical scenarios for seven different groups (Fig. S3). Within each group, demographic histories for two genetic clusters were estimated. As a control, demographic history within the genetic cluster I after dividing it into two population groups (I, I′) was additionally calculated. According to colonization routes after the LGM (Gugger et al. 2010), we expected the group I′ to be a post-Pleistocene (Holocene) founding derivative of the population group I. In general, three simple scenarios were simulated for all groups (Fig.4). In scenario 1, two populations *N*_1_ and *N*_2_ have diverged in the past from an ancestral population *N*_a_ and in scenario 2 and scenario 3, a classic invasion history was simulated where population A was derived from population B or vice versa, respectively. More complex histories could of course be simulated, but simplicity of the model is an important criterion when selecting among competing hypotheses (Knowles and Maddison 2002). We therefore also ignored the possibility that some populations may have undergone recent expansion.

For the coastal variety, the results of ABC analysis clearly favored the hypothesis that the population group I′ and genetic clusters II, III, IV, and V were derived from the cluster I as reflected in scenario 2 (Fig. S3). The posterior probabilities for this scenario ranged between 0.38 and 0.60 (95% CI 0.64– 0.34, Table S6). Two clear trends were obvious when connecting the divergence time to a period during the Pleistocene and Holocene. The divergence of coastal populations III, IV, V, and II from the coastal cluster I distributed in western Oregon and Washington was dated back to the Pleistocene before the LGM (Table S5) with average divergence time ranging from 40.1 to 56.9 ka ago (95% CI 197.0–10.5 ka). In contrast, the group I′ is a product of the Holocene and developed from cluster I around 5.37 ka BP (95% CI 12.2–1.78 ka). For the Rocky Mountain variety, genetic cluster VIII from the southern Rockies derived from VI covering northern and Canadian Rockies around 83.9 ka BP (95% CI 230.0–22.6 ka). The cluster VII situated in BC (Canadian Rocky Mountains) was estimated to be a derivate from cluster VI with a divergence time during Pleistocene around 41.7 ka ago (95% CI 143.0–7.48 ka). The posterior probabilities for both splits were 0.70 (95% CI 0.73–0.65) and 0.44 (95% CI 0.60–0.28), respectively (Table S6).

### Intervarietal hybridization and intervarietal introgression

In total, 25 individuals (with 0.2 < *Qvar *< 0.8) have been assigned to represent intervarietal-admixed genotypes by STRUCTURE (Fig.2). The majority of these genotypes (21) were identified in all four populations (R17, R18, R21, and R39) located in interior BC (Fig. S1), whereas in the populations R17 and R18 individuals of both varieties were also recognized (Fig. S1, Fig.2). The remaining four intervarietal-admixed individuals were identified in populations of coastal variety situated along slopes of the Cascades (R10, R15) in WA and in OR (R05) (Fig. S1). Within the Rocky Mountain variety, one such individual was found in the Blue Mountains of Oregon (R28). When extending the admixture boundaries (*Qvar*) for intervarietal-admixed individuals (0.15 < *Qvar *< 0.85), 12 admixed individuals could be additionally recognized within beforehand mentioned populations (R10, R17, R18, R28, and R39) with just one additional population R16 located close to R10. All are positioned in existing or possible contact regions between both varieties.

**Figure 5:**
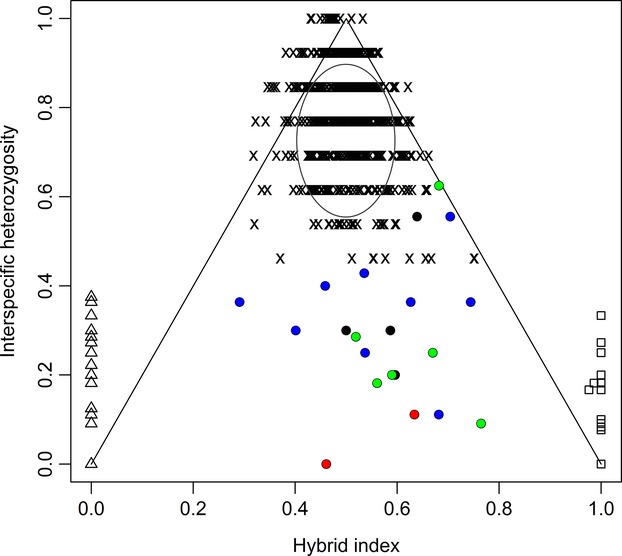
Intervarietal heterozygosity (*y*-axis) versus hybrid index (*x*-axis) estimated for variety-admixed genotypes from studied populations in British Columbia. Circles indicate observed values for variety-admixed individuals (red circles = R17, blue circles = R18, black circles = R21, green circles = R39); triangles indicate Rocky Mountain variety and tetragons stand for the costal variety. Crosses indicate values of simulated F1′s and the ellipse the 95% confidence intervals of simulated F1′s.

In the analysis of intervarietal heterozygosity (*IH*) versus maximum likelihood *HI*, the majority of the individuals with intervarietal-admixed genotypes from populations R17, R18, R21, R39 had a *HI* larger than 0.5 (*x*-axis in Fig.5). The intervarietal heterozygosities (*y*-axis) were distributed below the 95% confidence intervals of simulated F1′ intervarietal hybrids (Fig.5). The increase in the *HI* values of the majority of individuals with admixed genotypes moving toward *HI* values estimated for the coastal variety (0.9 < *HI* < 1) and their reduced *IH* pointed to their not recent origin and backcrossing to the coastal variety. Although both varieties might have come into contact at different times in Canada, there was no apparent pattern/grouping concerning *IH* and *HI* of these intervarietal recombinants and introgressants among populations (Fig.5).

## Discussion

### Divergence of coastal and Rocky Mountain varieties

The intervarietal divergence time as estimated by DIYABC was placed into mid-Pleistocene (708 ka, 95% CI 984–316 ka), a more recent period when compared to the older estimates of two recent reports based on cytoplasmic (cpDNA and mtDNA) data (Gugger et al. 2010; Wei et al. 2011). In the first estimate, Wei et al. (2011) dated the divergence of the coastal variety from the Rocky Mountain variety back to the Miocene 8.5 Ma (CI 16.5–2.4 Ma). The Miocene variety divergence was also in congruence with the paleobotanical evidence of both varieties (Critchfield 1984). The second estimate based on cytoplasmic data dated the intervarietal diversification to be more recent by placing it into Early Pleistocene 2.11 Ma (CI 4.37 Ma–755 ka) (Gugger et al. 2010). Although the intervarietal nuSSR estimates of this study overlapped with those of Gugger et al. (2010) and have even showed similarity to the mid-Pleistocene intervarietal split reported in an isoenzyme study (505–315 ka) (Li and Adams 1989), our nuSSRs based estimates for the intervarietal split must be interpreted with caution. Some possible explanations for the differing estimates compared to the two cytoplasmic DNA studies are dissimilarities in the mutation rate among these marker systems and different statistical models used. Furthermore, the rather recent nuSSR estimate of divergence was possibly affected (1) by the employment of a general and not Douglas-fir specific mutation rate for nuSSRs; (2) by the selection of only two genetic clusters for intervarietal calculations which may not cover the complex intervarietal divergence and most importantly, (3) by the fact that the algorithm used in DIYABC does not include any migration among varieties/clusters and may thus underestimate these estimates (Semerikov et al. 2013). Nevertheless, the nuSSR estimate may indicate an existing gene flow among varieties until the mid-Pleistocene. Similar to the post-Pleistocene colonization, secondary intervarietal contacts might have established during interglacials of the Pleistocene when both varieties might have periodically expanded and reduced their distribution range.

### Phylogeography of the coastal variety and refugia

In contrast to all previous studies, the nuSSRs revealed more detailed genetic structure of this variety by identifying five geographically distinct clusters (Table S6, Fig.1B). How does the distribution of these clusters and their divergence correspond to the location of fossil-determined glacial refugia and the postglacial colonization of this variety?

The allocation of the cluster I (refugial population 1) in western OR and WA was consistent with the existence of a former refugium on the Pacific coast of these states and with postglacial colonization to the south (California), to the east, where both slopes of the Cascades have primarily been colonized by it and to the north colonizing southern BC (Fig.1B). This glacial population, which all coastal clusters diverged from, even possessed the largest diversity indices of all clusters (Table S1A) as was also suggested in the refugia concept of Hewitt (1996).

In California, where fossil records indicated the other refugium (Gugger and Sugita 2010), not one but three refugial (glacial) populations were discovered: one along the coast (cluster IV), one near the Sierra Nevada (cluster III) and one in the north (cluster V) (Fig.1B); all with very similar Pleistocene divergence (56.9–40.1 ka, CI 197–10.1 ka) from the more northerly situated glacial population I (Table S6). Such detailed structuring has not yet been discovered in this area. However, rare alleles, different ecotypes, or a separate chemical race are acknowledged from other studies for these sites (Zavarin and Snajberk 1973, 1975; Klumpp 1999; Gugger et al. 2010). The location of the glacial population IV overlaps with Gugger et al.’s (2010) refugium located in the unglaciated San Francisco Bay area (Fig.1B). The glacial population III and V originated from other small-scale refugia. A similar phylogeographic pattern was revealed in *Notholithocarpus densiflorus* (tanoak), a forest tree associated with the Douglas-fir in California (Nettel et al. 2009). Our hypothesis on the existence of three refugial areas in California is built on larger genetic differentiation between III and IV when compared to III and V and IV and V (Table S3), their distances in the PCo (Fig.3C), genetic distances to cluster I, and the more ancient split of III from I (56.9 ka) than IV from I (45.9 ka) (Table S5). The range of topographic conditions in this area gives additional support for this reasoning (Fralish and Franklin 2002).

### Phylogeography of the Rocky Mountain variety and refugia

When comparing the distribution of the three nuSSR-based genetic clusters (VI–VIII, Fig.1B) to the location of the three refugia recognized by fossils (Gugger et al. 2010), then only the area of the southernmost cluster VIII with populations situated in Arizona, New Mexico and Colorado overlapped with a refugial area (Fig.1B). Identical to the coastal variety, these southern situated glacial populations have diverged from more northerly placed populations (cluster I of the coastal v., and cluster VI of the Rocky Mountain v.) in the Pleistocene. Similarly to this result, cpDNA types of Douglas-fir populations from the southern Rockies have been estimated to be of younger origin than those found in the central US Rockies (Wei et al. 2011). The northern US Rockies cluster VI, which spread into BC and from which both the southern (VIII) and the northern glacial population (VII) have diverged, has most probably originated from one of the two central US Rockies refugia, which were described by Gugger et al. 2010 (Fig.1B). The absence of material from these areas did not allow to clarify this.

### Possible cryptic glacial refugia inside and outside the current distribution range

The existence of one distinct glacial population for each variety (cluster II and VII) situated in the north of the current distribution area of Douglas-fir in BC (Fig.1B) led to the question from which refugia these northernmost populations in BC originated. Based on the geographic location of these populations and the divergence from the more southern refugial populations (I and VI) before the LGM (41.7 and 48.9 ka), we hypothesize that their origin is from two distinct cryptic and low-density refugia, which have escaped detection in the fossil record and thus have not been identified so far. These refugia may have been located either to the south of the Laurentide ice sheet (as the suggested Rocky Mountain refugium located in the northern US Rockies (Gugger et al. 2010)), but at higher latitudes than refugia for glacial populations I and VI or even in unglaciated areas located to the north of the ice sheet, in climatically favored microsites. Without backing from fossil evidence and an extensive sampling in Canada and the northern US Rockies, however, these populations cannot be attributed to either of them with confidence.

The existence of northern refugia situated to the north of the ice sheet, such in the Queen Charlotte Islands and in Beringia, has been inferred by paleorecords and/or suggested by genetic surveys for other temperate and boreal conifers co-occurring with Douglas-fir such as *Tsuga mertensiana* (Hansen and Engstrom 1996), *Pinus contorta* (Hansen and Engstrom 1996; Godbout et al. 2008; Strong 2010), *P. monticola* (Kim et al. 2011), *Picea sitchensis* (Gapare et al. 2005), *P. glauca* (Anderson et al. 2006, 2011), and *P. mariana* (Gérardi et al. 2010). Even for Douglas-fir and for the Rocky Mountain variety in particular, Wei et al. (2011) hypothesized on such possible northern refugium after the detection of an endemic cpDNA-type with unique and broad presence in BC. For the coastal variety, an own Douglas-fir ecotype classified by isoenzymes and geographic structure was found in the geographic area (the Chantslar lake, BC) of the northern cluster II (R38) (Klumpp 1999). If such northern refugia existed, then postglacial migration rates were even lower than their recent estimates (50–165 m/year, Gugger et al. 2010) and far below the migration rates required to keep up with future climate projections (1000 m/year, McLachlan et al. 2007).

### Intervarietal contact/hybrid zones and introgression

Canada was colonized by four genetically distinct populations, two of each variety (I, II, VI, VII) which expanded from four separate refugia. The postglacial colonization which brought both varieties to a contact at the present eastern border of the hybrid zone (reflected by the coexistence of both varieties within populations R17, R18) followed by intervarietal hybridization and predominant unidirectional introgression into the coastal variety rather than assortative mating of F1 hybrids (R17, R18, R21, and R39) led to a 450-km wide hybrid zone in BC (Gugger et al. 2010) (Fig.1A). A similar pattern was revealed using dominant markers by identifying an extensive and preferential pollen flow from east to the west of this hybrid zone, while seed-mediated gene flow remained geographically restricted (Gugger et al. 2010). In tree species, a high prevalence of introgressed genotypes over “true” hybrids is frequent and was also reported for other “Canadian” conifer hybrid zones such as for *Picea mariana* and *P. rubens* in northeastern Canada (Perron and Bousquet 1997), *Pinus contorta* and *P. banksiana* in Alberta in western Canada (Cullingham et al. 2012; Godbout et al. 2012), or *Picea sitchensis* and *P. glauca* in northern BC (Hamilton and Aitken 2013). Nevertheless, the structure of the Douglas fir hybrid zone in Canada is more complex than previously described. From the four populations (two of each variety) which colonized BC in the Holocene, three of them contributed to the intervarietal gene flow in addition to the intravarietal (intercluster) gene flow (cluster VI and VII with R18 and R21, Fig.1B). Further studies with more detailed sampling design is needed to investigate this complex situation in BC.

In addition to the hybrid zone described for Canada, the postglacial migration of both varieties led to two additional contact/transition zones in central Oregon and in the eastern Cascades of northern Washington. In central Oregon, when merging all published data (Li and Adams 1988, Gugger et al. 2010; Wei et al. 2011) including nuSSRs of this study, the transition zone covers an area from the eastern slopes of the Middle Cascades to the central part of the Blue Mountains in Oregon. In the second transition zone in the Cascades of WA, the Rocky Mountain variety spread into the coastal by pollen (Wei et al. 2011). An equivalent pattern of gene flow has been identified by SNPs (single nucleotide polymorphisms) associated with the cold-hardiness of the Douglas-fir growing east of the Cascade crest in Washington. Despite the possible recent intervarietal contact in the Cascades, cold-adapted alleles which originated and introgressed from the Rocky Mountain variety have been discovered in individuals of the coastal variety (Eckert et al. 2009).

## Conclusion and Outlook

The codominant SSRs markers allowed novel insights into the Quaternary glacial epoch dynamics of this tree species including (1) the estimation of glacial history and divergence times for six distinct genetic clusters (populations), (2) the identification of so far the most comprehensive phylogeographic structure of the coastal variety, and finally, (3) the revealing of a complex postglacial recolonization in BC.

Existing phylogeographic patterns allow light to be shed on the impact of past climatic cycles on species distributions, which in turn may help to facilitate predictions on how species will respond to future climates. Recent distribution models which incorporated intraspecific variation (taken to be presence of different subspecies within a species, or provenance trial data) resulted in a different (and more optimistic) projection of future suitable habitats than the models that treated species as single entities (e.g., Oney et al. 2013). Such projections may differ even more when incorporating more details such as glacial and postglacial populations and the hybridization events as revealed in this study.

And finally, the Douglas-fir is only one of several broadly distributed and economically important forest tree species (such as *Abies grandis, Picea sitchensis*, *Pinus contorta*, *Robinia pseudoacacia*, *Quercus rubra)* with extensive introduction outside their native ranges, where they sometimes naturalize or even become invasive (Call and Nilsen 2005; Kota et al. 2007; Traveset et al. 2008; Cierjacks et al. 2013; Radtke et al. 2013). Douglas-fir was introduced in numerous countries worldwide (e.g., New Zealand, Chile, Argentina, Australia, France) (Bastien et al. 2013). In European forestry, this conifer is seen as an alternative to some native tree species mainly because of its stable growth potential even under the dryer conditions, which are predicted for the future (IPCC 2007; Eilmann et al. 2013). Detailed knowledge of the genetic structure and hybridization/introgression patterns of this tree in northern America is crucial (1) for understanding how this species may evolve in artificially variety-mixed stands existing in Europe (Fussi et al. 2013) and (2) for planning for future climates and stable production while considering that imported seeds from areas in interior BC and along the eastern Cascades in OR and WA are likely to be of intervarietal-admixed character and therefore not “pure” varieties.

## Data Archiving Statement

Sampling locations: supplementary material.

Microsatellite genotypic data will be submitted in STRUCTURE format to Dryad.

## Supporting Information

**Figure S1.** Distribution map of Douglas-fir populations (R01-39) within its natural range in Northwest America. **Figure S2.** Plots of Principal Coordinates analysis (Pcoa) described by two principal coordinates (PCo1, PCo2) and explained genetic variance (%) for populations (R01-R39) with inter-varietal-admixed individuals and individuals of the opposite variety (A) for the entire data set, (B) for the coastal variety (C) and the Rocky Mountain variety. **Figure S3.** Different scenarios arranged into 7 groups to analyse the demographic history of the coastal and Rocky mountain variety. **Table S1A.** Population number (Pop.-Nr.), geographic origin (state-prov., and coordinates inclusive altitude), number of collected individuals (N), number of mother trees (NM): mostly 15 (Kleinschmidt and Bastien 1992) (A), >20 (B), >50 (C), ∼ 50 (D), unknown (E), 15 from 5 stands (F), 15 (G). **Table S1B.** Genetic cluster (C) membership (I–IX based on STRUCTURE), population number (Pop. -Nr.), variety assignment (*Qvar*), intra-varietal assignment (*Qintra*) to the genetic cluster (C) marked in bold, heterozygosities *H*o and *H*E, allelic richness (*A*s), inbreeding coefficent (*F*IS). **Table S2.** nuSSRs loci, their multiplex combination (1–4), and used (optimized) annealing temperature. **Table S3.** Genetic variability at 13 nuclear microsatellite in coastal and Rocky-Mountain Douglas-fir, including number of alleles (*Na*) mean number of alleles (*A*), effective number of alleles (*N*e), observed heterozygosity (*H*O), expected heterozygosity (*H*E), inbreeding coefficient (*F*IS), allelic richness (*A*s). **Table S4.** Pairwise Jost’s D values among all nine genetic Douglas-fir clusters based on 12SSRs (without locus 5A8) below diagonal. **Table S5.** Estimations of the posterior distributions of parameters revealed from the Approximate Bayesian Computation for the best scenario. **Table S6.** Posterior probabilities for the scenarios with the highest posterior probability of 10,000 set of summary statistics most similar to the observed data through logistic regression. **Table S7.** Populations of each cluster/group for DIYABC.
