# Supplementary material for: Intervarietal and intravarietal genetic structure in Douglas-fir: nuclear SSRs bring novel insights into past population demographic processes, phylogeography, and intervarietal hybridization
Source: Ecol Evol. 2015 Apr 3;5(9):1802–17. doi: 10.1002/ece3.1435 (PMC4485962; doi:10.1002/ece3.1435)

Supplementary material

Table S1A: Population number (Pop.-Nr.), geographic origin (state-prov., and coordinates inclusive altitude), number of collected individuals (N), number of mother trees (N^M): mostly 15 (Kleinschmidt & Bastien 1992) (A), >20 (B) , >50 (C) , ~ 50 (D), unknown (E), 15 from 5 stands (F), 15 (G).

Pop. -Nr.	State-Prov.	Lat.	Long.	Alt.[m]	N	N^M
R01	US-OR	44.41	122.47	525	20	B
R02	US-OR	44.22	122.07	975	20	B
R03	US-OR	45.77	123.22	200	20	A
R04	US-OR	44.50	122.00	975	20	A
R05	US-OR	45.40	121.38	975	20	B
R06	US-OR	45.38	122.30	250	20	A
R07	US-WA	46.37	123.73	45	18	A
R08	US-WA	47.25	123.42	100	20	A
R09	US-WA	45.97	121.53	675	20	B
R10	US-WA	46.04	121.44	1125	20	B
R11	US-WA	46.75	122.13	525	20	B
R12	US-WA	48.30	121.60	825	20	B
R13	US-WA	48.26	121.56	375	20	B
R14	US-WA	48.65	121.72	475	20	A
R15	US-WA	47.54	121.55	525	20	B
R16	US-WA	46.50	121.89	525	20	B
R17	CA-BC	49.50	117.27	825	20	B
R18	CA-BC	51.17	119.54	525	20	B
R19	CA-BC	49.00	121.75	475	20	A
R20	US-WA	48.60	118.73	750	20	B
R21	CA-BC	52.69	122.43	650	20	A
R22*	US-AZ	34.93	111.35	2200	20	E*
R23*	US-NM	33.43	108.60	2500	20	E*
R24*	US-NM	32.83	105.55	2400	20	E*
R25*	US-NM	35.75	105.83	2700	20	E*
R26*	US-CO	37.95	105.07	3200	20	E*
R27*	US-ID	46.47	115.35	900	20	E*
R28*	US-OR	44.95	118.15	1125	20	E*
R29*	US-CA	40.85	123.43	100	20	E*
R30*	US-OR	44.38	123.88	225	22	E*
R32*	CA-BC	49.10	124.03	450	20	E*
R33***	US-MT	46.99	110.70	1800	20	E
R34***	US-CA	37.92	120.05	975	20	F
R35***	US-CA	39.87	122.67	825	20	A
R36***	US-CA	40.36	121.83	975	22	E
R37***	US-CA	40.14	124.05	825	22	G
R38**	CA-BC	52.35	126.03	450	22	D
R39**	CA-BC	54.04	125.34	850	20	C

* Populations provided by Klumpp, ** provided by the BC Forest Service and *** provided by the USDA Forest Service, Placerville Nursery.

Table S1B: Genetic cluster (C) membership (I-IX based on STRUCTURE), population number (Pop. -Nr.), variety assignment (*Qvar*), intra-varietal assignment (*Qintra*) to the genetic cluster (C) marked in bold, heterozygosities H_o and H_E, allelic richness (*A*s), inbreeding coefficent (F_{IS}).

Pop. - Nr.	State-Prov.	*Qvar*	Genetic Cluster	*Qintra*	H_o	H_E	*A*s	F_{IS}
R03	US-OR	0.99	**I**	0.82	0.712	0.883	6.3	0.22[1]
R07	US-WA	0.98	**I**	0.83	0.711	0.880	6.1	0.23[1]
R08	US-WA	0.99	**I**	0.83	0.677	0.880	6.2	0.26[1]
R10	US-WA	0.94	**I**	0.82	0.634	0.881	6.2	0.31[1]
R11	US-WA	0.98	**I**	0.92	0.753	0.904	6.5	0.20[1]
R12	US-WA	0.99	**I**	0.85	0.688	0.870	6.1	0.24[1]
R13	US-WA	0.99	**I**	0.82	0.678	0.886	6.3	0.26[1]
R15	US-WA	0.98	**I**	0.89	0.711	0.887	6.4	0.23[1]
R16	US-WA	0.97	**I**	0.90	0.691	0.889	6.3	0.25[1]
R19	CA-BC	0.98	**I**	0.83	0.653	0.884	6.2	0.29[1]
				**Overall**	**0.696**	**0.924**	**6.4**	0.25
R01	US-OR	0.99	**I**-(III+IV+V)	0.72	0.710	0.893	6.4	0.23[1]
R02	US-OR	0.99	**I**-(III+IV+V)	0.73	0.687	0.891	6.4	0.26[1]
R04	US-OR	0.99	**I**-(III+IV+V)	0.68	0.720	0.890	6.5	0.22[1]
R05	US-OR	0.98	**I**-(III+IV+V)	0.75	0.670	0.891	6.4	0.28[1]
R06	US-OR	0.99	**I**-(III+IV+V)	0.73	0.672	0.864	6.2	0.25[1]
R09	US-WA	0.99	**I**-(III+IV+V)	0.77	0.716	0.886	6.3	0.22[1]
R14	US-WA	0.99	**I**-(III+IV+V)	0.78	0.681	0.869	6.0	0.25[1]
R29	US-CA	0.95	**I**-(III+IV+V)	0.58	0.705	0.891	6.5	0.24[1]
R30	US-OR	0.98	**I**-(III+IV+V)	0.68	0.685	0.900	6.5	0.26[1]
R38	CA-BC	0.98	**II**	0.91	**0.669**	**0.826**	**5.4**	**0.23[2]**
R32	CA-BC	0.99	**I**-II	0.64	**0.699**	**0.866**	**6.0**	**0.22[1]**
R34	US-CA	0.99	**III**	0.89	**0.656**	**0.820**	**5.4**	**0.23[2]**
R35	US-CA	0.97	**IV**	0.82	**0.694**	**0.851**	**5.7**	**0.22[1]**
R36	US-CA	0.98	**V**	0.99	0.640	0.866	6.1	0.29[1]
R37	US-CA	0.95	**V**	0.92	0.678	0.888	6.3	0.26[1]
				**Overall**	**0.665**	**0.901**	**0.28[2]**	**6.5**
R20	US-WA	0.04	**VI**	0.85	0.506	0.841	5.9	0.44[1]
R27	US-ID	0.06	**VI**	0.92	0.593	0.880	6.3	0.36[1]
R28	US-OR	0.06	**VI**	0.88	0.545	0.853	5.9	0.40[1]
R33	US-MT	0.04	**VI**	0.92	0.547	0.858	6.1	0.40[1]
				**Overall**	**0.557**	**0.908**	**6.4**	0.40
R17	CA-BC	0.14	**VI**-VII	0.62	0.561	0.857	5.8	0.39[1]
R18	CA-BC	0.37	**VI**-VII	0.68	0.543	0.884	6.4	0.43[1]
R21	CA-BC	0.13	VI-**VII**	0.73	0.607	0.870	6.1	0.34[1]
R39	CA-BC	0.19	**VII**	0.96	**0.618**	**0.805**	**5.4**	**0.27[2]**
R22	US-AZ	0.01	**VIII**	0.99	0.633	0.735	5.7	0.17[1]
R23	US-NM	0.01	**VIII**	0.95	0.562	0.730	5.7	0.26
R24	US-NM	0.01	**VIII**	0.98	0.491	0.629	5.7	0.25[1]
R25	US-NM	0.02	**VIII**	0.94	0.624	0.808	6.0	0.29[1]
R26	US-CO	0.01	**VIII**	0.89	0.517	0.754	6.0	0.35[1]
				**Overall**	**0.581**	**0.803**	**5.9**	**0.29[2]**

[1]Significant deficit of heterozygotes for populations, based on 9880 randomizations. P-value was less than or equal to the indicative adjusted nominal level (5%) of 0.0001.
[2]Significant deficit of heterozygotes for clusters, based on 117000 randomizations. P-value was less than or equal to the indicative adjusted nominal level (5%) was 0.00043.

Table S2: nuSSRs loci, their multiplex combination (1-4), and used (optimized) annealing temperature.

Locus	multiplex combination	Annealing temperature
2G12	1	54°C
4A7	1	
3B2	1	
5A8	1	
2D4	2	50°C
1F9	2	
3F1	2	
3B9	3	50°C
3D5	3	
2D9	3	
2D6	4	57°C
1C3	4	
2C2	4	

Table S3: Genetic variability at 13 nuclear microsatellite in coastal and Rocky-Mountain Douglas-fir, including number of alleles (*Na*) mean number of alleles (*A*), effective number of alleles (*N*e), observed heterozygosity (H_O), expected heterozygosity (H_E), inbreeding coefficient (F_{IS}), allelic richness (*A*s).

		Na	***A***	***Ne***	H_O	H_E	F_{IS}	***As***
**1C3**	coastal	43	14.4 ± 0.583	11.3 ± 0.435	0.540 ± 0.027	0.907 ± 0.005	**0.43** ± 0.032	9.8
	interior	43	14.2 ± 0.861	9.9 ± 0.894	0.602 ± 0.035	0.883 ± 0.017	**0.34** ± 0.050	6.9
**1F9**	coastal	62	20.2 ± 0.476	13.7 ± 0.492	0.827 ± 0.020	0.924 ± 0.030	**0.13** ± 0.022	10.3
	interior	75	20.9 ± 0.953	14.3 ± 1.178	0.747 ± 0.038	0.924 ± 0.007	**0.22** ± 0.046	7.3
**2C2**	coastal	30	11.2 ± 0.517	6.4 ± 0.459	0.753 ± 0.024	0.821 ± 0.015	**0.11** ± 0.024	7.1
	interior	16	5.4 ± 0.646	3.7 ± 0.425	0.473 ± 0.039	0.681 ± 0.036	**0.32** ± 0.077	4.4
**2D4**	coastal	45	18.0 ± 0.532	12.7 ± 0.547	0.720 ± 0.023	0.916 ± 0.005	**0.24** ± 0.025	9.9
	interior	41	14.6 ± 1.118	11.5 ± 0.800	0.557 ± 0.066	0.908 ± 0.007	**0.42** ± 0.073	7.1
**2D6**	coastal	55	15.9 ± 0.681	11.8 ± 0.586	0.614 ± 0.016	0.909 ± 0.005	**0.35** ± 0.018	10.3
	interior	38	5.7 ± 0.711	4.1 ± 0.795	0.219 ± 0.047	0.583 ± 0.087	**0.54** ± 0.106	4.8
**2D9**	coastal	49	14.7 ± 0.528	7.5 ± 0.464	0.770 ± 0.027	0.854 ± 0.009	**0.12** ± 0.033	8.1
	interior	59	15.9 ± 1.190	13.3 ± 0.809	0.496 ± 0.053	0.922 ± 0.005	**0.49** ± 0.059	7.3
**2G12**	coastal	34	13.6 ± 0.494	8.4 ± 0.412	0.810 ± 0.021	0.874 ± 0.006	**0.10** ± 0.021	8.2
	interior	56	19.8 ± 1.297	13.9 ± 1.225	0.827 ± 0.029	0.921 ± 0.007	**0.13** ± 0.028	7.3
**3B2**	coastal	50	16.8 ± 0.525	11.2 ± 0.557	0.651 ± 0.020	0.904 ± 0.006	**0.30** ± 0.023	9.9
	interior	34	10.1 ± 0.851	5.1 ± 0.525	0.596 ± 0.044	0.764 ± 0.034	**0.26** ± 0.045	5.5
**3B9**	coastal	46	15.1 ± 0.644	10.1 ± 0.588	0.767 ± 0.022	0.889 ± 0.010	**0.16** ± 0.025	8.9
	interior	50	16.2 ± 0.912	10.0 ± 0.752	0.837 ± 0.018	0.893 ± 0.008	**0.09** ± 0.021	7.0
**3D5**	coastal	32	13.1 ± 0.523	10.0 ± 0.454	0.559 ± 0.023	0.893 ± 0.007	**0.40** ± 0.028	9.0
	interior	61	18.6 ± 1.470	13.3 ± 1.295	0.814 ± 0.034	0.915 ± 0.010	**0.14** ± 0.036	7.2
**3F1**	coastal	43	15.5 ± 0.444	10.6 ± 0.381	0.735 ± 0.022	0.903 ± 0.004	**0.21** ± 0.024	9.2
	interior	61	10.9 ± 1.152	9.7 ± 0.996	0.345 ± 0.042	0.832 ± 0.070	**0.62** ± 0.035	7.4
**4A7**	coastal	63	17.1 ± 0.536	11.7 ± 0.519	0.667 ± 0.027	0.910 ± 0.004	**0.29** ± 0.031	10.1
	interior	39	9.9 ± 0.678	5.2 ± 0.819	0.580 ± 0.043	0.764 ± 0.030	**0.26** ± 0.062	5.4
**5A8**	coastal	13	4.7 ± 0.189	3.5 ± 0.136	0.525 ± 0.016	0.703 ± 0.012	**0.29** ± 0.022	4.6
	interior	9	3.5 ± 0.685	3.0 ± 0.582	0.255 ± 0.065	0.516 ± 0.102	**0.58** ± 0.069	*
**Over-all**	coastal	43	14.6 ± 0.247	9.9 ± 0.198	0.688 ± 0.008	0.877 ± 0.004	**0.24** ± 0.009	8.9
	interior	47	12.7 ± 0.497	9.0 ± 0.395	0.565 ± 0.019	0.808 ± 0.016	**0.34** ± 0.051	6.5

Significant deficit of heterozygotes for populations, based on 2600 randomizations. Loci in boldface have a p-value less than or equal to the indicative adjusted nominal level (5%) of 0.00192.

Table S4: Pairwise Jost's D values among all nine genetic Douglas-fir clusters based on 12SSRs (without locus 5A8) below diagonal. Probability based on 999 permutations is shown above diagonal. The grey highlighted cells represent the Rocky Mountain variety.

I	II	III	IV	V	VI	VII	VIII	
	0.001	0.001	0.001	0.001	0.001	0.001	0.001	I
0,432		0.001	0.001	0.001	0.001	0.001	0.001	II
0,515	0,580		0.001	0.001	0.001	0.001	0.001	III
0,459	0,572	0,561		0.001	0.001	0.001	0.001	IV
0,301	0,445	0,349	0,463		0.001	0.001	0.001	V
0,631	0,760	0,751	0,726	0,642		0.001	0.001	VI
0,636	0,712	0,700	0,762	0,690	0,566		0.001	VII
0,739	0,825	0,779	0,839	0,786	0,407	0,667		VIII

Table S5: Estimations of the posterior distributions of parameters revealed from the Approximate Bayesian Computation for the best scenario. Estimation is based on 1% of the closest simulated data sets and the logit transformation of parameters was used.

			N_1	N_2	*t1*	*t2*	N_a
Group 1	I - III	Average	8.36×10^{3}	1.04×10^{3}	5.69×10^{4}	5.16×10^{5}	4.91×10^{3}
		Median	8.56×10^{3}	9.16×10^{2}	4.30×10^{4}	4.99×10^{5}	4.78×10^{3}
		Mode	9.78×10^{3}	8.72×10^{2}	2.61×10^{4}	4.03×10^{5}	2.76×10^{3}
		95% CI	5.78×10^{3}	3.64×10^{2}	1.16×10^{4}	8.72×10^{4}	4.43×10^{3}
			9.94×10^{3}	2.62×10^{3}	1.97×10^{5}	9.73×10^{5}	9.64×10^{3}
Group 2	I - IV	Average	8.04×10^{3}	1.95×10^{3}	4.59×10^{4}	5.88×10^{5}	5.00×10^{3}
		Median	8.20×10^{3}	1.62×10^{3}	3.61×10^{4}	6.09×10^{5}	4.89×10^{3}
		Mode	9.33×10^{3}	1.37×10^{3}	1.93×10^{4}	8.22×10^{5}	4.17×10^{3}
		95% CI	5.13×10^{3}	6.04×10^{2}	1.05×10^{4}	1.02×10^{5}	4.99×10^{2}
			9.91×10^{3}	5.74×10^{3}	1.42×10^{5}	9.79×10^{5}	9.69×10^{3}
Group 3	I - V	Average	8.02×10^{3}	4.03×10^{3}	4.01×10^{4}	5.11×10^{5}	4.97×10^{3}
		Median	8.18×10^{3}	3.61×10^{3}	3.41×10^{4}	4.97×10^{5}	4.85×10^{3}
		Mode	8.45×10^{3}	3.31×10^{3}	2.00×10^{4}	1.69×10^{5}	3.21×10^{3}
		95% CI	5.27×10^{3}	1.23×10^{3}	1.06×10^{4}	7.53×10^{4}	5.30×10^{2}
			9.86×10^{3}	8.97×10^{3}	1.05×10^{5}	9.72×10^{5}	9.60×10^{3}
Group 4	I – I′	Average	8.52×10^{3}	3.16×10^{3}	5.37×10^{3}	4.50×10^{5}	5.15×10^{3}
		Median	8.68×10^{3}	2.68×10^{3}	4.82×10^{3}	4.23×10^{5}	5.07×10^{3}
		Mode	9.23×10^{3}	2.05×10^{3}	4.16×10^{3}	8.52×10^{4}	3.91×10^{3}
		95% CI	6.14×10^{3}	7.21×10^{2}	1.78×10^{3}	3.57×10^{4}	5.61×10^{2}
			9.93×10^{3}	8.58×10^{3}	1.22×10^{4}	9.62×10^{5}	9.68×10^{3}
Group 5	I - II	Average	8.37×10^{3}	1.17×10^{3}	4.89×10^{4}	5.21×10^{5}	4.58×10^{3}
		Median	8.56×10^{3}	1.02×10^{3}	3.71×10^{4}	5.15×10^{5}	4.27×10^{3}
		Mode	9.40×10^{3}	9.07×10^{2}	2.80×10^{4}	2.23×10^{5}	2.34×10^{3}
		95% CI	5.75×10^{3}	3.94×10^{2}	9.35×10^{3}	8.61×10^{4}	3.66×10^{2}
			9.93×10^{3}	2.82×10^{3}	1.55×10^{5}	9.74×10^{5}	9.62×10^{3}
Group 6	VI - VII	Average	7.72×10^{3}	8.92×10^{2}	4.17×10^{4}	5.32×10^{5}	6.87×10^{3}
		Median	7.87×10^{3}	7.09×10^{2}	3.01×10^{4}	5.28×10^{5}	7.38×10^{3}
		Mode	8.23×10^{3}	6.26×10^{2}	1.65×10^{4}	2.96×10^{5}	8.95×10^{3}
		95% CI	4.69×10^{3}	2.63×10^{2}	7.48×10^{3}	8.50×10^{4}	1.34×10^{3}
			9.90×10^{3}	2.78×10^{3}	1.43×10^{5}	9.75×10^{5}	9.88×10^{3}
Group 7	VI-VIII	Average	8.03×10^{3}	4.10×10^{3}	8.39×10^{4}	4.89×10^{5}	5.39×10^{3}
		Median	8.19×10^{3}	3.76×10^{3}	7.02×10^{4}	4.60×10^{5}	5.45×10^{3}
		Mode	8.20×10^{3}	3.25×10^{3}	4.89×10^{4}	3.27×10^{5}	5.93×10^{3}
		95% CI	5.18×10^{3}	1.32×10^{3}	2.26×10^{4}	7.99×10^{4}	7.24×10^{2}
			9.89×10^{3}	8.95×10^{3}	2.30×10^{5}	9.66×10^{5}	9.67×10^{3}

N_1 and N_2 indicate effective population of cluster analysed, N_a effective population size of ancestral population, *t1* and *t2* time since divergence in years.

Table S6: Posterior probabilities for the scenarios with the highest posterior probability of 10 000 set of summary statistics most similar to the observed data through logistic regression.

			posterior probability	95% CI
Group 1	I – III	Scenario 2	0.5613	0.5162 – 0.6064
Group 2	I – IV	Scenario 2	0.5959	0.5558 – 0.6360
Group 3	I – V	Scenario 2	0.3848	0.3381 – 0.4316
Group 4	I – I′	Scenario 2	0.4145	0.3759 – 0.4531
Group 5	I – II	Scenario 2	0.4351	0.3952 – 0.4756
Group 6	VI – VII	Scenario 2	0.6918	0.6494 – 0.7341
Group 7	VI - VIII	Scenario 2	0.4370	0.2806 – 0.5934

Table S7: Populations of each cluster/group for DIYABC.

I	R01 – R11, R30
I`	R12 – R14, R19, R32
II	R38
III	R34
IV	R35
V	R36, R37
VI	R17, R20, R27, R28, R33
VII	R39
VIII	R22 – R26

Figure S1: Distribution map of Douglas-fir populations (R01-39) within its natural range in Northwest America.

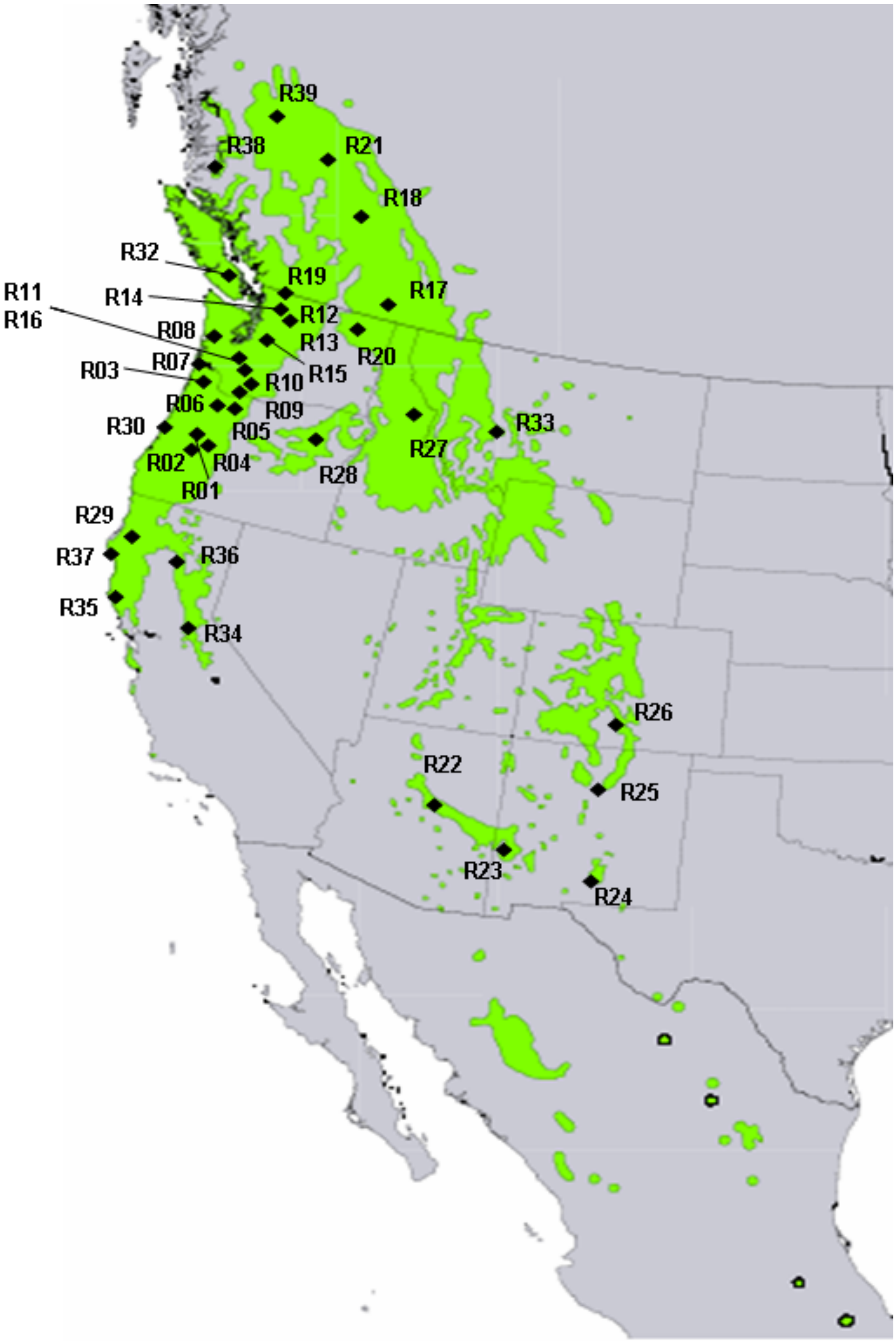

Figure S2: Plots of Principal Coordinates analysis (Pcoa) described by two principal coordinates (PCo1, PCo2) and explained genetic variance (%) for populations (R01-R39) with inter-varietal-admixed individuals and individuals of the opposite variety (A) for the entire data set, (B) for the coastal variety (C) and the Rocky Mountain variety. Roman numbers (in parentheses) at the back of each population represent a membership to the particular genetic cluster as assigned by STRUCTURE analysis. Populations of identical STRUCTURE cluster are grouped together (solid circles). The cluster- admixed populations are embodied by half filled dots. Dots with missing population numbers represent populations of the cluster I and its cluster-admixed populations.

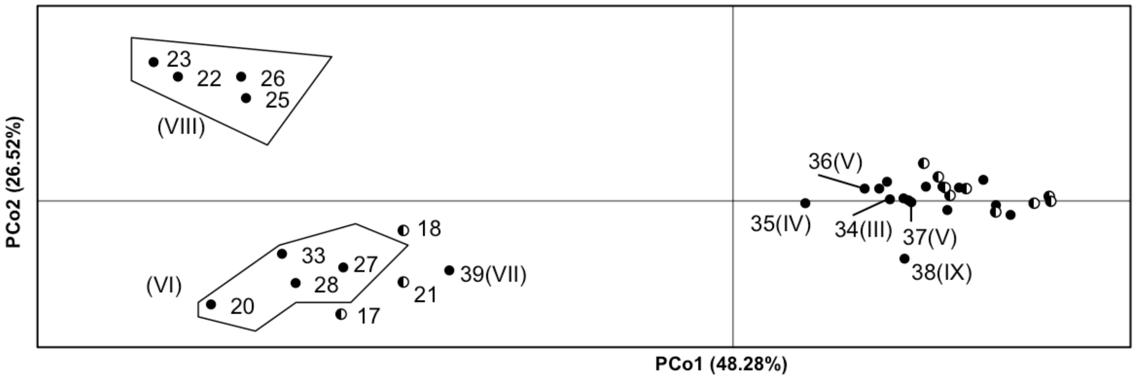

(A)

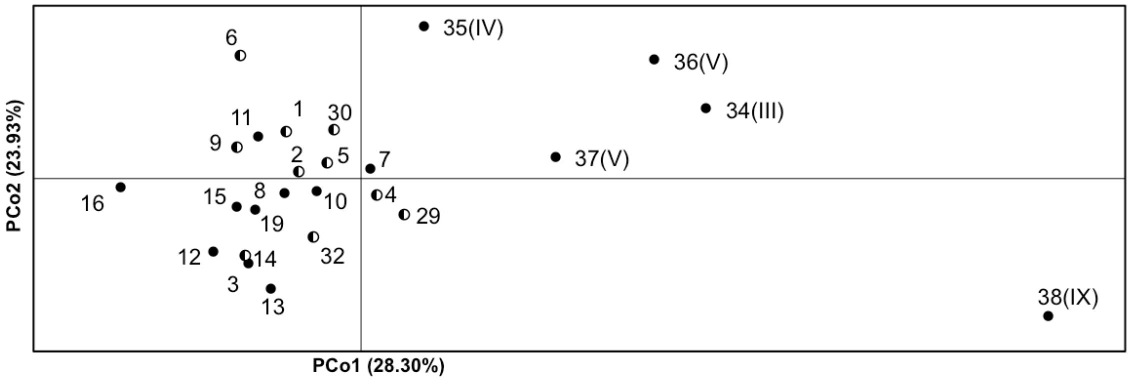

(B)

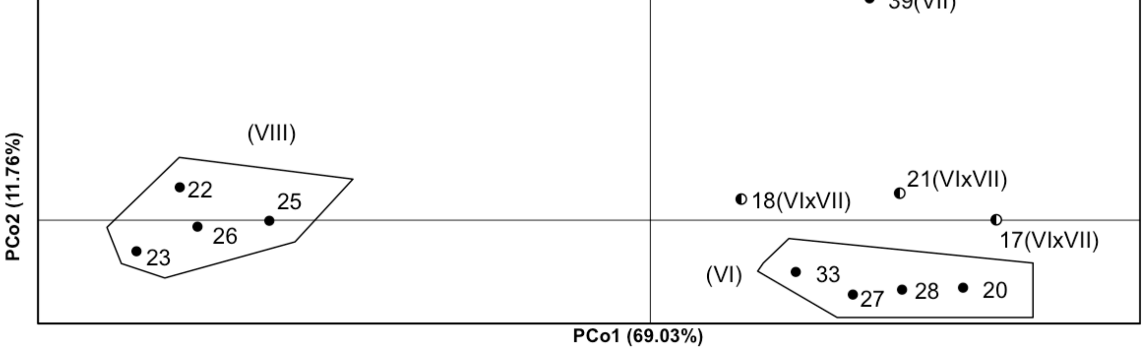

(C)

Figure S3: Different scenarios arranged into 7 groups to analyse the demographic history of the coastal and Rocky mountain variety. A * indicate the scenario with the highest posterior probabilities.

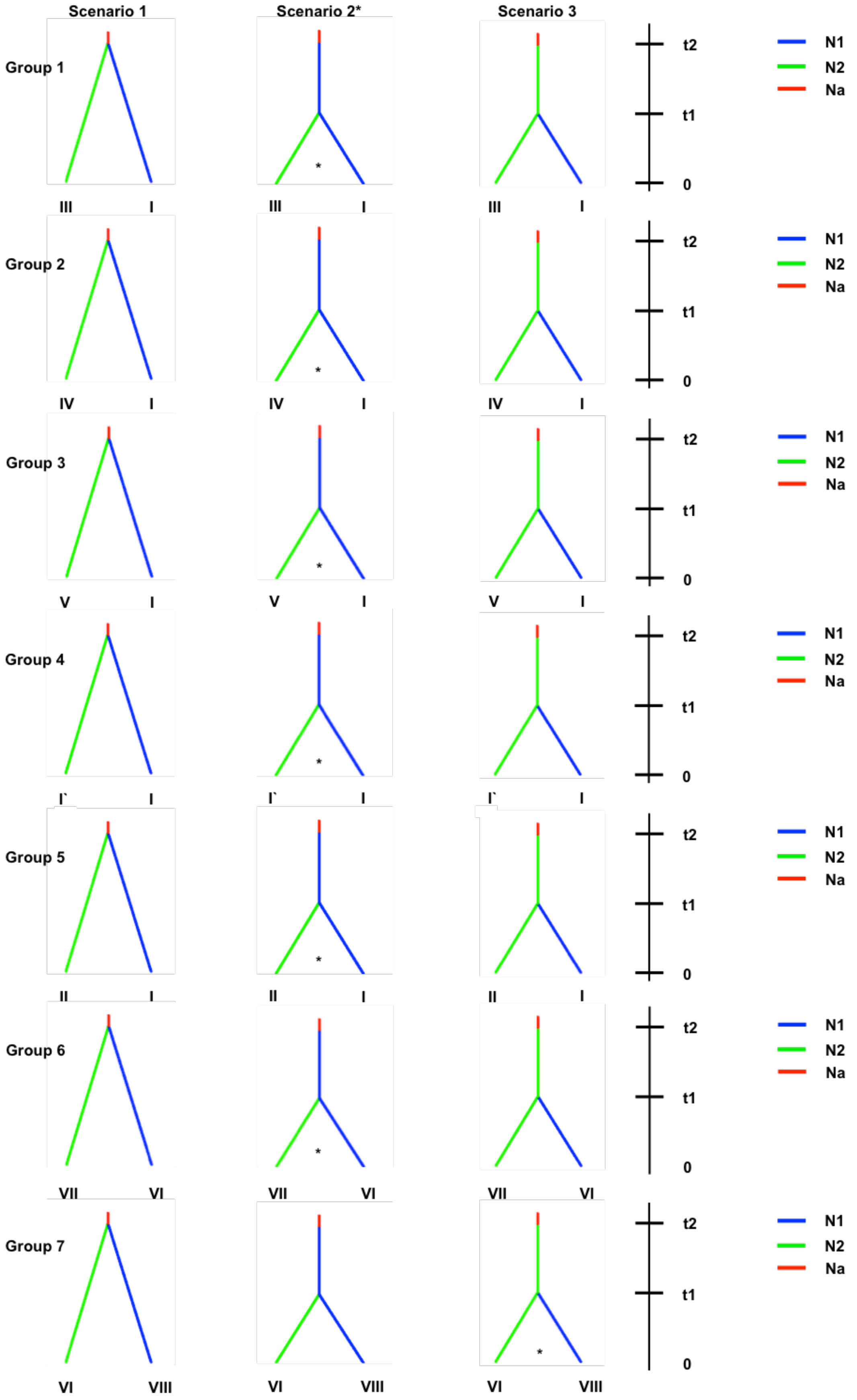

Supplement: Supplementary file 1 [file ece30005-1802-sd1.pdf]
